# Energy Regulation in Inflammatory Sarcopenia by the Purinergic System

**DOI:** 10.3390/ijms242316904

**Published:** 2023-11-29

**Authors:** Miguel Marco-Bonilla, Maria Fresnadillo, Raquel Largo, Gabriel Herrero-Beaumont, Aránzazu Mediero

**Affiliations:** Bone and Joint Research Unit, IIS-Fundación Jiménez Díaz UAM, 28040 Madrid, Spain; miguel.marcob@quironsalud.es (M.M.-B.); maria.fresnadillo@quironsalud.es (M.F.); rlargo@fjd.es (R.L.); gherrero@fjd.es (G.H.-B.)

**Keywords:** adenosine, muscle, sarcopenia

## Abstract

The purinergic system has a dual role: the maintenance of energy balance and signaling within cells. Adenosine and adenosine triphosphate (ATP) are essential for maintaining these functions. Sarcopenia is characterized by alterations in the control of energy and signaling in favor of catabolic pathways. This review details the association between the purinergic system and muscle and adipose tissue homeostasis, discussing recent findings in the involvement of purinergic receptors in muscle wasting and advances in the use of the purinergic system as a novel therapeutic target in the management of sarcopenia.

## 1. Management of Energy Distribution in the Body

Energy homeostasis is defined as the regulation of energy utilization for essential physiological processes and the production of compounds. It revolves around maintaining a balance between energy intake and expenditure to sustain bodily functions and weight [1]. Energy expenditure refers to the amount of energy an individual uses to maintain essential bodily functions such as breathing, circulation, and digestion [2].

The energy generated by the metabolism is primarily derived from glucose. Glucose is converted to ATP as immediate energy needed for many essential processes in organisms and cells [3,4]. Following hydrolysis of its phosphate groups, ATP releases its storage energy and provides energy for cells [5,6]. The efficiency of ATP formation is only about 43%, and the remaining 57% is lost as heat in the body’s metabolism [7].

The relationship between body composition and energy metabolism has been the subject of research for many years. Keesey and Hirvonen demonstrated that energy requirements increase proportionally with bodyweight in rats [8]. Even at rest, there is a baseline energy consumption associated with organs with high metabolic rates [9]. The rate of energy consumption for each organ depends on its individual metabolism and size, and this dynamic process can vary with growth, the onset of diseases, and nutritional status. It is known that under resting conditions, skeletal muscle, heart, liver, brain, kidneys, and adipose tissue exhibit the highest basal metabolic rates [9].

During an illness, the basal metabolic rate can vary dramatically depending on the type, severity, and stage of the disease [10]. In untreated inflammatory conditions, the immune system is the primary energy-consuming organ, accounting for 10–15% of the total energy expenditure [11]. Chronic, low-grade systemic inflammation, characterized by increased pro-inflammatory cytokines, such as obesity, cachexia, or exercise, also results in significant energy expenditure. In exercise, interleukin 6 (IL-6) myokine acts as an energy sensor, triggering lipolysis and ATP generation as muscle glycogen is consumed during contraction [12,13]. On the other hand, IL-6 produced by macrophages in obesity and aging causes an increase in lipolysis and high levels of free fatty acids [11].

Caloric restriction is the most commonly employed method for weight loss. While it can yield initial results, prolonged caloric restriction often leads to diminished effectiveness in achieving weight loss. During caloric restriction, the first organs to reduce their metabolism are skeletal muscle and adipose tissue, whereas the remaining organs and tissues tend to be largely preserved [3].

## 2. The Double Edge of the Purinergic System

In 1929, Albert Szent-Gyorgyi and Alan Drury proved that purines and pyrimidines are involved in extracellular signaling [4]. However, it was not until 1972 when Geoffrey Burnstock showed that ATP was a transmitter in non-adrenergic and non-cholinergic inhibitory nerves that purinergic signaling was proposed [14]. Adenosine and ATP play a dual role within the purinergic system. They mediate both energy storage and release through ATP and their phosphate hydrolysis to ADP and AMP, thereby meeting cellular energy demands and facilitating nucleotide assembly. Additionally, they serve as signaling molecules [15].

The purinergic system mediates cell signaling through the activation of selective receptors (named purinergic receptors) and secondary pathways for the control of physiological actions (such as cell proliferation/differentiation) [16]. Purinergic receptors are divided into two groups (based on agonist selectivity), namely P1 adenosine receptors and P2 nucleotide receptors. Two subfamilies of P2 receptors (ionotropic P2X receptors and metabotropic P2Y receptors) [16,17] and four different P1 receptor sub-types (A1R, A2AR, A2BR, and A3R) have been characterized [18]. P1 receptors are activated at different adenosine concentrations, where A1R and A2AR are high-affinity receptors (<1 µM adenosine) and A2BR and A3R are low-affinity receptors (<10 µM adenosine) [19].

The sequential hydrolysis of extracellular ATP to adenosine is catalyzed by ectonucleotidases (CD39, CD73). Once adenosine is produced, it is released via cell membrane equilibrative (ENT) and concentrative (CNT) nucleoside transporters. Adenosine release via ENT keeps intracellular and extracellular adenosine levels in balance, while CNT favors intracellular adenosine levels [20]. Extracellular adenosine can either be converted to inosine by adenosine deaminase [21] or it can activate adenosine receptors. Moreover, intracellular adenosine can sequentially be converted to AMP, ADP, and ATP via phosphorylation or into uric acid as a final metabolite [17].

All adenosine receptors belong to the GPCR family of seven transmembrane receptors linked to calcium mobilization, either promoting (A2AR/A2BR) or inhibiting (A1R/A3R) the generation of cyclic AMP (cAMP) [22]. A1R and A3R are negatively coupled to adenylate cyclase, while A2AR and A2BR are positively coupled to adenylate cyclase [23]. The inhibition of cAMP due to A1R and A3R stimulation has been shown to promote the contraction of smooth muscle cells via MAPK and ERK1/2 activation [24]. Increased cAMP levels lead to the activation of protein kinase A (PKA) and the exchange protein directly activated by cAMP (EPAC) [25]. PKA and EPAC may perform individual, combination, or opposite effects [25,26]. Studies in cancer and neuronal models have shown that PKA activation promotes proliferation, while EPAC activation promotes differentiation [26]. Furthermore, EPAC is able to promote osteoclast formation, and PKA counteracts this effect [27,28]. In contrast, in vitro smooth muscle models have shown that both PKA and EPAC promote muscle proliferation [29]. Therefore, the role of these cAMP receptors is tissue dependent. The activation of the cAMP response element binding protein (CREB) is triggered by the phosphorylation of PKA in the nucleus. CREB phosphorylation has been related to the activation of pro-anabolic genes [30] and the reduction in pro-atrophic genes in muscle [31].

ATP is released to the extracellular space via connexins (e.g., connexin-43) and pannexin channels (Panx-1, Panx-2, Panx-3) [15,17,23]. In addition, some experiments show that connexins are not clearly expressed in adult muscles. Consequently, the process of releasing ATP to the extracellular space might be carried out by pannexin1 hemichannels [32]. The physiological effects of elevated extracellular ATP are mediated by P2X and P2Y receptors. P2X receptors are ligand-gated ion channels, while P2Y receptors are members of the G protein-coupled receptor (GPCR) family. There are seven sub-types of P2X receptor (1–7) and eight sub-types of P2Y receptors (P2Y1, P2Y2, P2Y4, P2Y6, P2Y11–14), all of which are selective for ATP, ADP, UTP, and UDP [4,17,33].

Moreover, the purinergic system exerts its role to fulfill cellular energy demands via modulation of the AMP-activated protein kinase (AMPK)-mediated mechanism. AMPK is a ubiquitously distributed serine/threonine protein kinase that regulates cellular energy homeostasis, acting as a central energy sensor and maintaining energy stores by fine-tuning anabolic and catabolic pathways through the activation of pathways that generate ATP (e.g., glucose transport and glycolysis) and the deactivation of energy-consuming anabolic pathways (e.g., inhibition of fatty acid synthesis) [34]. AMPK is activated via the phosphorylation of tyrosine residue 172 with the increase in the intracellular AMP–ATP ratio [35]. Moreover, creatine kinase (CK) mediates an energetic role in the purinergic system. ATP generated via mitochondrial oxidative phosphorylation is used to catalyze the conversion of creatine (Cr) to phosphocreatine (PCr) [36]. Then, PCr is released into the cytosol and activates CK to regenerate the ATP consumed during muscle expenditure [36]. Elevated levels of PCr in the cytosol are related to inactive AMPK due to high ATP concentrations [37]. Therefore, AMPK and CK act as mediators of energy control in the purinergic system [38]. Alterations in the energy balance between anabolism and catabolism generate a poor metabolic rate, which leads to wasting conditions, such as aging [39].

## 3. Sarcopenia

Sarcopenia is a generalized and progressive loss of skeletal muscle mass and function, concomitant with an increased risk of adverse outcomes such as falls, metabolic dysfunction, disability, poor quality of life, and death [40,41]. After the age of 30, an individual loses between 3 and 8% of muscular mass every decade, with this rate increasing after the age of 60 [42]. This decrease in muscle mass produces a decline in strength and muscular function, with qualitative changes in muscular tissue due to a reduction in motor units affecting both nervous and muscular fibers, especially fiber type II, and, therefore, altering the contractile activity [43,44].

Sarcopenia can be categorized as primary or secondary sarcopenia. Primary sarcopenia is associated with the aging process, seen in the elderly, and secondary sarcopenia is associated with one or more of the following causes that could promote the loss of muscle mass: sedentary lifestyle, immobilization, malnutrition, diabetes, obesity, cancer, and other acute or chronic inflammatory diseases (e.g., rheumatoid sarcopenia) [45,46]. The European Working Group on Sarcopenia in Older People (EWGSOP) described the following criteria for the diagnosis of sarcopenia: low skeletal muscle mass (diagnosed via DXA or anthropometry) and either low muscle performance (walking speed, muscle power) or low muscle strength (e.g., handgrip) [47,48].

**Figure 1:**
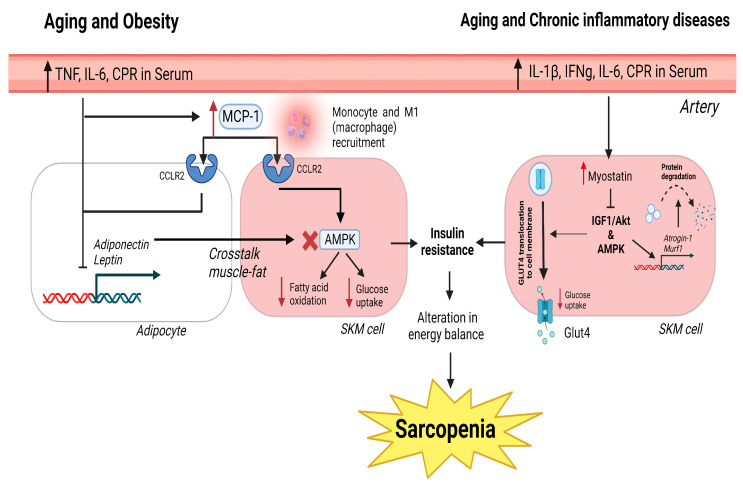
Canonical pathways in obesity, aging, and chronic inflammatory diseases that generate sarcopenia. In obesity and aging, the increase in pro-inflammatory cytokines and CRP in the serum generates high levels of MCP-1, which binds to the CCLR2 receptor in muscle and adipose cells. In adipose tissue, this causes the inhibition of adiponectin and leptin expression. The inhibition causes a negative cross-talk between muscle and the inhibition of AMPK, fatty acid oxidation, and glucose uptake. In chronic inflammatory disorders and aging, the presence of pro-inflammatory molecules in the serum produces an increase in myostatin. Myostatin inhibits IGF1/Akt via AMPK activation. This causes a reduction in glucose uptake and an increase in atrogene expression underlying protein degradation and muscle atrophy. The inhibition of glucose uptake in obesity, aging, and chronic inflammatory disorders generates insulin resistance and muscle loss due to the failure to restore muscle cell energy demands. Red cross indicates blockade of the expression of AMPK. Red arrows inform of up or down regulation of which is indicated next to it.

In addition, when considering the duration, sarcopenia can be described as acute sarcopenia when lasting less than 6 months and chronic sarcopenia when the duration is longer than 6 months, and it is associated with other chronic conditions, including aging [49] (Figure 1).

Although sarcopenia has a severe impact on the quality of life of elderly people and society, the pathophysiological mechanisms underlying this disease have not been elucidated due to the intricacy of the network of interactions and causes (chronic inflammation, muscle protein turnover alterations, neuromuscular junction dysfunction, and hormone levels, among others) [50]. Muscle loss in both primary and secondary sarcopenia appears to be driven by different mechanisms, involving changes in biochemical molecules in different signaling pathways [51,52]. Sarcopenia in the elderly is primarily caused by anabolic resistance induced by myostatin [51], while secondary sarcopenia appears to be activated by catabolic processes [52]. In this regard, the severity of secondary sarcopenia observed in chronic debilitating conditions and inflammatory diseases varies with the intensity of systemic inflammation [53].

Low-grade chronic inflammation, produced by slight elevations in circulating pro-inflammatory mediators (such as C-reactive protein (CRP), tumor necrosis factor (TNF), and IL-6), is among the causes of inducing sarcopenia. Catabolic inflammatory processes are involved in the development of sarcopenia, especially at an advanced age, even in healthy individuals [54]. This low-grade chronic inflammation presents immune cell senescence, alterations in T cell function and the extracellular matrix, foci of chronic infection, and an increased fat mass [55]. Recent data from sarcopenic elderly individuals suggest that circulating TNF and IL-6 are significantly elevated, and these circulating levels are correlated with and increased risk of muscle strength loss [56,57]. Under normal circumstances, CRP, TNF, and IL-6 maintain the balance between catabolism and synthetic metabolism in skeletal muscles, but higher levels of inflammatory markers are associated with physical decline, resulting in increased catabolism, with an inhibition of protein synthesis, and damage in muscle integrity and function, resulting in sarcopenia [58,59]. On the other hand, IL-6 has been proven to act as a sensor of muscle damage, promoting the activation and migration of T cells via gap junction proteins, such as connexins and pannexins [60]. Anti-inflammatory cytokines (IL-4, IL-10, and IL-15) are able to antagonize pro-inflammatory cytokine activity to reduce muscle atrophy and retard sarcopenia [61]. It has been observed that IL-4 improves glucose metabolism in muscle cells and acts as a myoblast recruitment factor, promoting myogenesis and muscle regeneration [62,63] (Figure 1).

Frailty is a multi-system syndrome associated with lower resilience against stressors and an increased risk of adverse health outcomes [64,65]. Both low muscle strength or function and weight loss are phenotypic characteristics of the frailty syndrome [66], reflecting that frailty and sarcopenia are linked, although they are distinct, as not all frailty in patients is related to skeletal muscle mass or function [64]. Both conditions share the same pathophysiology and clinical outcomes, with sarcopenia being considered a component of frailty, but not vice versa [67]. Therefore, diagnostic criteria are essential for the recognition of each condition in clinical practice. According to Fried et al., individuals can be categorized as non-frail (0 Fried criteria present), pre-frail or intermediate (1–2 criteria), or frail (≥3 criteria) [64], with these criteria observing the following factors: low gait speed and low grip strength, weight loss, self-reported low physical activity, and exhaustion [66]. Sarcopenia is then considered the physical component of frailty. As described above, it is clear that the degree and chronicity of inflammation are responsible for the effect on muscle mass, strength, and quality. Relatively mild inflammation levels that occur in normal aging or obesity may not be sufficient to observe the effects on muscle mass or strength lost but affect metabolic quality and, therefore, contribute to sarcopenia development [68]. In a more severe systemic inflammation scenario, as seen in frailty, pro-inflammatory cytokines contribute to muscle mass and strength loss [69]. Exogenous TNF administration to mice induced anorexia and muscle loss, the upregulation of leptin, activation of NFκB (nuclear factor kappa B), atrophy and activation of the ubiquitin–proteasome pathway, and suppression of the AKT serine–threonine protein kinase and mammalian target of rapamycin (Akt-mTOR) pathway [70]. Moreover, neutrophils and NET (neutrophil extracellular traps) generation are impaired with age [71]. Higher neutrophil counts have been associated with frailty and low levels of physical activity, and higher white cell count in healthy individuals can predict frailty over the years [72]. The neutrophil chemotactic ability is reduced with age, inducing an inefficient migration, higher tissue damage, and secondary systemic inflammation, suggesting that neutrophils play a key role in sarcopenia and frailty [67,73].

Rheumatoid arthritis (RA) is the most common autoimmune disease, with 1% of the world population affected [74]. Apart from articular manifestations, RA has several systemic comorbidities, including rheumatoid cachexia (RC), a condition that impacts function and quality of life and affects approximately 11% to 26% of RA patients worldwide [51,75]. It is characterized by a reduced skeletal muscle mass with either stable or increased fat mass, degradation of balance and disruption of muscle protein synthesis, decreased muscle mass, strength, and function, together with total energy expenditure, insulin resistance, increased basal metabolic rate, and inflammation [51]. The mechanism involved is not fully understood but includes cytokine-driven hypermetabolism produced by TNF, IL-1β, and IL-6 (associated with resting energy expenditure and sarcopenia in RA patients), as well as the limitation of physical activity and insulin resistance [51,76]. Novel approaches indicate that insulin resistance is produced in RA patients due to the increase in energy expenditure via high levels of IL-6 in the serum [77].

Employing an antigen-induced arthritis animal model, we have previously demonstrated that rabbits developed a rheumatoid cachexia-like secondary sarcopenia with increased muscle protein breakdown and a compensatory anabolic response [51]. Rheumatoid rabbits showed weight loss, decreased muscle size, and an upregulation of atrogenes in muscles, along with a decrease in myostatin expression and a reduction in the signal transducer and activator of transcription 3 (p-STAT-3) levels. This response suggests that the inflamed muscle could contribute to secondary sarcopenia through an autocrine mechanism of atrophy triggered by pro-inflammatory mediators [51]. In an adjuvant-induced arthritis model, the authors have observed a 20% decrease in skeletal muscle and muscle weight [78]. In a collagen-induced arthritis (CIA) rat model, Hartog et al. have described reduced weight and spontaneous locomotion. Additionally, a 31% reduction in the gastrocnemius relative weight has also been demonstrated 21 days after the induction of arthritis [78,79]. Numerous studies have monitored alterations in body composition parameters and, consequently, the development of rheumatoid cachexia (RC). This was carried out through the implementation of specific drug treatments or a combination of therapeutic approaches. Disease-modifying anti-rheumatic drugs (DMARDs) play a crucial role in managing disease activity by impeding inflammatory signaling pathways, such as those involving TNF and IL-6. Methotrexate (MTX) monotherapy is a first line DMARD agent for RA. It is unknown whether MTX monotherapy is beneficial for RC, but some authors have demonstrated that MTX in combination with different drugs may protect against the development of RC [75]. The use of Janus kinase (JAK) inhibitors has also been explored. The IL-6/JAK/STAT pathway is key for muscle fiber development and regeneration. Multiple studies have demonstrated that the JAK/STAT pathway controls the myogenic development of adult satellite cells [80]. Furthermore, the JAK/STAT pathway induced the expression of atrogenes Murf1 and Atrogin1 in muscle alterations derived from RA [81]. In an antigen-induced arthritis rabbit model of RA, the inhibition of the JAK/STAT pathway with tofacitinib prevents the expression of the atrogenes and myogenic alterations in muscles [81].

Sarcopenia is sometimes accompanied by changes in adipose tissue. The modulation of myokine and adipokine levels contributes to the cross-talk between muscle and adipose tissue [82]. Secretions of these cytokines regulate anabolic and catabolic responses in muscles. These cytokines are altered with high adiposity and age-related muscle wasting [82]. Myostatin and irisin are the main myokines involved in the muscle–fat cross-talk [83,84]. Myostatin is a human growth factor that produces a downregulation of protein synthesis in muscles via Smad2/3 and inhibits insulin-like growth factor-1 (IGF-1)/Akt via (forkhead box transcription factors) FOXO; moreover, it inhibits glucose transporter protein type-4 (GLUT4) and AMPK [85,86]. This pathway produces a reduction in glucose uptake and leads to muscle atrophy. With aging, myostatin expression increases with a strong correlation to decreased strength [87]. Clinical studies have shown that patients with obesity have an increased presence of myostatin in the serum [88]. Therefore, there is a relationship between fat and myostatin expression that has not been elucidated (Figure 1).

In contrast, irisin correlates with increased strength and muscle maintenance via Akt/mTor [89]. The activation of this pathway has been demonstrated in a C2C12 model, in which irisin treatment was observed to contribute to the development of muscle hypertrophy [90,91]. In addition, a decrease in irisin expression was found in patients with severe obesity [92]. Therefore, there is a relationship that has not been elucidated between fat and the expression of both myostatin and irisin. On the other hand, among the adipokines involved in the muscle–fat relationship are leptin and adiponectin. Leptin is known to be a pro-inflammatory adipokine. It is closely related to the amount of body fat and acts on muscle via the modulation of AMPK levels [93]. Old obese rats show resistance to leptin. On the other hand, the caloric restriction of these models increases responsiveness to leptin (especially in aging) [94]. In humans, serum leptin levels were positively correlated with body mass index (BMI) and negatively correlated with skeletal muscle index (SMI) [95]. This indicates that leptin is a good marker to indicate the risk of sarcopenic obesity [95].

Adiponectin is a key regulator synthesized by adipose tissue involved during glucose and fatty acid metabolism. Adiponectin promotes the ability of insulin to stimulate glucose uptake through increased GLUT4 translocation to the plasma membrane. C2C12 myoblast cells transfected with adiponectin showed reduced lipid accumulation [96]. The role of adiponectin in sarcopenia is unclear, but clinical studies have shown that the level of adiponectin is significantly lower in sarcopenic patients [97]. However, a parallel clinical study observed that strength was negatively correlated with adiponectin expression [98].

The principal function of monocyte chemoattractant protein-1 (MCP-1/CCL2) is to regulate monocyte/macrophage migration and infiltration [99]. Increased adipose tissue has been shown to correlate with increased MCP-1, which promotes macrophage migration into the adipose tissue and the synthesis of other cytokines, such as IL-6 and TNF [100,101,102]. Cytokine expression has been studied in patients with cachexia, and only MCP-1 was found to be increased, indicating a major role in muscle loss [103]. This was confirmed in a clinical study, where sarcopenic patients showed increased serum MCP-1 expression [104]. Therefore, MCP-1 favors a pro-inflammatory state in adipose and muscle tissues, leading to the development of sarcopenia (Figure 1).

## 4. P2 Receptors in Muscle and Fat in Sarcopenia

The presence of P2 purinergic receptors has been proven in muscles using immunochemistry. P2Y11 and P2X1 are more expressed in the cytosol of muscle fibers. In addition, P2X1 is expressed in the plasma membrane, as well as P2Y4. In contrast, other receptors, such as P2Y1, P2Y2, P2Y12, and P2X4, are not expressed in muscle cells [105]. In sarcolemma, receptors P2X1 and P2Y4 are visible. P2X1 is found inside vesicles in sarcolemma and P2Y4 does not have these vesicles. The P2Y11 receptor is expressed in type I fibers, while it is less expressed in type 2 fibers and almost absent in sarcolemma [105].

P2 receptors are correlated with muscle blood flow. Experiments with the continuous infusion of ATP have been shown to stimulate muscle blood flow, allowing us to prove that P2 receptors are involved during physical exercise in muscles. This was proven using a P2Y1R blocker. It has been shown that upon blocking this receptor, the potentiation (contraction) in the EDL muscle was prevented, whereas it was not prevented in the soleus. This information suggests that ATP is able to activate P2Y1 receptors in fast muscles [32]. On the other hand, the use of P2 receptor inhibitors led to a decrease in muscle blood flow [106]. In addition, the use of ATP increases the abundance of NA^+^–K^+^ pumps in muscles. This means that P2 receptors are involved in muscle excitation during intense exercise [107].

Additionally, chronic inflammatory diseases that generate muscle dystrophies produce an increase in the amount of ATP in muscle tissue [107]. This ATP activates the P2X7 receptor, which has been correlated with muscle fiber atrophy [108]. Experiments in a co-culture with osteoclast and muscle cells showed that the mechanical stimulation of osteoclast releases ATP in the medium. This ATP initiates a cross-talk with muscles, inducing the P2–PI3K–Akt-mTOR pathway in muscles [109]. On the other hand, knockout models of P2X7 showed a reduction in the expression of CCL2 and IL6 in WAT, but no alterations were observed in BAT thermogenesis [110]. In adipocytes, it has been observed that the expression of P2X7 is related to an inhibition of SIRT3/5 genes involved in browning, favoring the adipogenesis process [111]. Therefore, it is possible that the expression of P2X7 generates muscle atrophy, increases adipogenesis, and reduces BAT, contributing to the development of obese sarcopenia in chronic inflammation.

The action of P2Y receptors is controversial. P2Y1 and P2Y2 receptors improve muscle regeneration, which is blocked when these receptors are inhibited [112]. However, in skeletal muscle fibroblasts, P2Y2 receptors promote fibrosis and muscle atrophy via Akt/ERK/PKC activation [113]. Therefore, the role of P2Y receptors in the development of sarcopenia remains unknown.

## 5. Adenosine Receptors in Muscles and Fat and Their Role in Sarcopenia

The expression and distribution of adenosine receptors in skeletal muscle may change depending on the species, and within the species, it may depend on the muscle state, type of muscle fiber, and location within the fiber. In vitro, in the C2C12 myoblastic line, A1R was first characterized, correlating its expression with the cellular enhancement of glucose uptake [114]. A subsequent study analyzed the gene expression of all receptors in C2C12, in which A2BR was the most highly expressed. A1R, A2AR, and A3R were expressed to a lesser extent compared to A2BR [115]. In mouse tibialis, the expression of A1R, A2AR, and A2BR was observed via a polymerase chain reaction (PCR) [115,116]. Moreover, in rats, the expression of these receptors was also confirmed via Western blotting [117]. In human skeletal muscle, the expression of A2AR and A2BR in the plasma membrane and cytosol and the absence of A1R were verified via immunohistochemistry. A2AR is equally distributed in type 1 and 2 fibers and is slightly more expressed in the cytoplasm than in the membrane of type 1 fibers. In contrast, A2BR has major expression in the cytoplasm of type 2 and the plasma membrane of type 1 fibers. Therefore, according to the expression of the receptors in the cell membrane in comparison with cytosol, A2AR is responsible for glucose transport across the cell membrane in type 2 fibers and A2BR in type 1 fibers [118]. When blocking adenosine receptors or using adenosine deaminase to remove extracellular adenosine, there is a great decrease in glucose transport in skeletal muscle fibers, which confirms their role in glucose metabolism [119].

**Figure 2:**
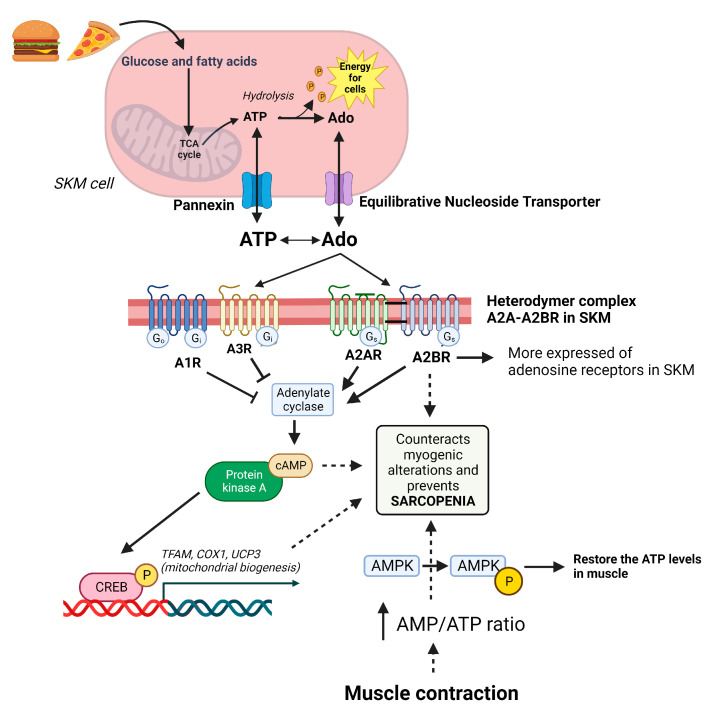
Purinergic system in the management of muscle homeostasis. Fatty acids and glucose are used to produce cell energy via ATP and are recovered via adenosine. ATP and adenosine maintain a balance in the cytosol and extracellular space via pannexin and an equilibrative nucleoside transporter, respectively. In muscles, the A2B receptor (the most expressed of the adenosine receptors in skeletal muscle (SKM)) forms a heterodymer with the A2A receptor to exert its function. These receptors activate cAMP/PKA/CREB, which increases the mitochondrial biogenesis to maintain energy balance in the muscle cell. On the other hand, muscle contraction uses available ATP. This produces an increase in the AMP/ATP ratio and activates AMPK, which is able to restore ATP levels in the cell. Therefore, A2BR, cAMP, and AMPK are essential molecules in the control of muscle homeostasis and future pharmacological targets in the treatment of sarcopenia.

The activation of PKA and AMPK depends on the expression of adenosine A2A and A2B receptors [120,121]. The upregulation of A2A and A2B promotes the formation of cyclic AMP from ATP by adenylate cyclase [122]. This cAMP activates both PKA and exchange proteins directly activated by cyclic AMP (EPAC) proteins. Next, cAMP is reduced to AMP by phosphodiesterase [123]. This increases the intracellular AMP concentration and thus the AMP/ATP ratio, similar to events during muscle contraction [124]. This ratio activates AMPK via phosphorylation and allows for the restoration of ATP levels from AMP [125]. The activation of both PKA and AMPK has been observed to promote CREB expression [121,126,127]. CREB promotes the expression of genes involved in mitochondrial biogenesis (PGC-1 and TFAM) and muscle regeneration, preventing alterations in muscles [128,129] (Figure 2).

Adenosine receptors differ in expression within adipose tissue depending on whether they are located in white (WAT) or brown (BAT) adipose tissue. Vassaux et. al. analyzed the mRNA expression of adenosine receptors in the white adipocytes and pre-adipocytes extracted from rat epididymal white fat pad. A2R expression was characterized in pre-adipocytes but not adipocytes, and A1R was only in adipocytes [130,131]. However, in studies on mesenchymal cell differentiation to adipocytes, it has been observed that both A1R and A2AR are more expressed with the passage of days of adipocyte differentiation [132]. On the other hand, in murine BAT, the expression of the four adenosine receptors was demonstrated [115]. There is some controversy because some studies suggest that A2AR is the most abundant adenosine receptor in human and murine BAT [133,134]. However, subsequent studies have shown a predominant role of A2BR in murine BAT [115]. This may be due to the heterodimerization of A2AR–A2BR, in which the expression of both is required for activation [115]. Methotrexate treatment in RA increases the adenosine available to adenosine receptors, decreases joint inflammation, and induces the browning of adipose tissue with a high expression of genes that induce thermogenesis in RA [135].

In a first study, treatment with a cAMP analog (db-cAMP) was associated with an extended average lifespan and the maintenance of muscle mass, thus preventing the occurrence of sarcopenic events in mice [136]. The production of cAMP in skeletal muscle is primarily dependent on A2BR [115,137]. An A2BR^−/−^ (skeletal muscle-specific knockout) murine model led to a loss of muscle mass and strength and increased senescence markers and mitochondrial alteration [115]. In addition, in skeletal muscle explants from patients, a higher expression of A2BR was correlated with a lower expression of the senescence marker p21 [115]. In primary myocytes isolated from these same patients and treated with an A2BR agonist, an increase in differentiation and proliferation markers was observed, as well as an improvement in the expression of mitochondrial oxidative phosphorylation markers [115].

Because the development of sarcopenia is accompanied by an increase in fat in what is known as obese sarcopenia, it is important to establish the role of adenosine receptors in both WAT and BAT [138,139]. Adenosine is secreted from adipocytes, activating the A1R, which is involved in multiple functions. In isolated rat adipocytes, increased A1R expression has been correlated with increased lipolysis [140]. On the other hand, increased A2BR expression appears to inhibit lipogenesis and adipogenesis [141]. In human and murine brown adipocytes, adenosine activates its receptors at nanomolar levels. The inhibition of A2AR, both pharmacologically and in a murine A2Aknockout model, has shown that A2A is an essential contributor to the process of thermogenesis [133]. Subsequently, it was proven in the A2BR murine knockout model that the activation of this receptor with an agonist increased the thermogenesis process and decreased the induction of obesity via diet [115]. The aforementioned evidence seems to lead to the conclusion that both A2AR and A2BR (as heterodymers) make an essential contribution to preventing the onset of the sarcopenic process and the development of obesity correlated with muscle loss [115].

## 6. Therapeutic Possibilities: Adenosine in Tissue Regeneration

The main role of adenosine is to maintain cellular homeostasis and it is of special interest as a target in the treatment of many diseases and disorders [6]. Clinical treatment with adenosine is not very effective due to its short lifetime and receptor non-specificity [142]. However, several approaches have been developed for the therapeutic use of the purinergic system, including the oral or intravenous administration of ATP, the use of AR agonists, the use of adenosine analogs or drugs that modulate cellular levels of adenosine and increase its selectivity toward an AR, and the use of a cAMP analog [143].

In relation to muscle disorders, some therapeutic strategies correlated with the purinergic system were investigated.

### 6.1. A2B Signaling via AMPK/cAMP and Mitochondrial ADP Sensibility Counteracts Aging Sarcopenia

AMPK activation has been shown to inhibit the progression of aging via FoxO, mTOR, CREB, and sirtuin (SIRT) 1 signaling pathways [144]. AMPK activation has been correlated with increased levels of cAMP via Ca^2+^/calmodulin-dependent protein kinase II (CaMKII) [145,146]. Because the increase in cAMP in muscles primarily depends on A2BR, an A2BR agonist could be a good therapeutic agent to counteract muscle loss in aging [115]. In addition, adenosine has been shown to prevent age-related loss of muscle contraction [147,148]. In a parallel study, CaMKII has been shown to decrease muscular fatigue by reducing calcium release during intense exercise [149]. CaMKII also promotes ATP signaling via P2R and pannexin, contributing to the migration of dendritic cells during muscular damage [150]. Furthermore, the commitment of myoblasts to the myogenic lineage relies on an increase in intracellular free calcium levels. Potential cell membrane pathways implicated in these calcium increases include P2 receptors and connexin and/or pannexin hemichannels, recognized for their ability to allow for the passage of calcium [151].

A downregulation of A2BR in aging was observed. Studies on the A2BR receptor agonist BAY 60-6583 in rats have demonstrated an improvement in muscle contraction [147]. The increase in cAMP as a molecule to counteract sarcopenia has not only been proven by A2BR stimulation but also by using a cAMP analog (db-cAMP) [136]. Muscle cAMP levels decreased in 24-month-old mice compared to 6-month-old mice, concomitant with a loss of motor activity that was recovered with db-cAMP treatment [136]. On the other hand, AMPK activation depends on the intracellular AMP/ATP ratio, and an increase in AMP levels using a pharmacological modulator is a potential strategy to address sarcopenia in aging [152]. Alternatively, ADP sensitivity has been shown to be reduced in old mouse gastrocnemius, with an increased production of reactive oxygen species (ROS) [153]. This increase in ROS leads to an age-associated increase in H_2_O_2_ release [154]. Insulin use has been shown to contribute to increased ADP sensitivity in mitochondria [155]. As such, insulin may be a good stimulating agent of mitochondrial biogenesis via ADP in aging.

Recently, it was reported that the pharmacological increase in extracellular adenosine by dipyridamole in the myoblastic line C3C12 leads to an increase in A2B adenosine receptor expression [127]. Subsequently, this leads to cAMP–PKA–CREB increase and AMPK activation [127]. Furthermore, the pharmacological stimulation of cAMP and AMPK by dipyridamole is able to prevent alterations in muscle myogenesis in vitro [127]. Therefore, a new therapeutic via the treatment of sarcopenia is introduced with the use of drugs that modulate adenosine levels in muscles (e.g., dipyridamole) [127]. On the other hand, the use of tenofovir in the C2C12 line inhibits ATP release in the extracellular space [127]. This produces a decrease in extracellular adenosine levels, with a reduced expression in the adenosine A2B receptor [127]. The decrease in the adenosine 2B receptor promotes alterations in muscle myogenesis with the inhibition of PKA/AMPK pathways [127].

### 6.2. cAMP Treatment to Prevent Muscle Atrophy

Muscle atrophy is characterized by alterations in protein metabolism, leading to a loss of muscle function [156]. AMP deaminase 3 controls the content of intracellular adenine nucleotides. AMP deaminase 3 has been shown to be increased in a murine skeletal muscle atrophy model [157]. The overexpression of AMP deaminase 3 is related toa decrease in ATP and an increase in inosine monophosphate (IMP) levels [157]. Furthermore, the upregulation of AMP deaminase 3 produces an inhibition of AMPK phosphorylation and decreases in the mitochondrial protein synthesis rate [157]. In this case, the stimulation of AMP production by an analog may be a good therapeutic approach to avoid muscle wasting in atrophic muscles. In addition, AMPK activity is decreased in the extensor digitorum longus of atrophic rats [158]. Therefore, implementing a cAMP analog, as mentioned previously, could increase the intracellular AMP/ATP ratio, thereby preventing AMPK inactivation [152].

### 6.3. ATP as a Therapy for Cancer-Associated Cachexia

Cancer-associated cachexia occurs in half of all cancer patients [159]. The level of muscle loss varies with the progression and type of tumor; therefore, maintaining muscle mass is essential to improve the quality of life and treatment efficacy [160]. Patients with gastric cancer have a lower content of ATP, ADP, AMP, and adenosine [161]. This decrease in purines and pyrimidines is not due to a lack of nutrients. This was proven in the muscle of a cancer cachexia model in pair-fed rodents, in which tumor resection increased ATP levels [162,163]. Intravenous ATP has already been safely tested in lung cancer patients. Both in phase I and phase II studies, ATP has shown promising results in muscle maintenance and nutritional status [164]. In murine models, it has been observed that intraperitoneal ATP inhibits weight loss in animals with advanced tumor growth independent of its antineoplastic action [165]. On the other hand, prolonged oral use of ATP (0 to 5000 mg/day) has been shown to lead to a decrease in ATP plasma levels in biodistribution studies, which decreases its therapeutic potential [166].

### 6.4. Adenosine Modulators as a Treatment for Duchenne Muscular Dystrophy

Duchenne muscular dystrophy (DMD) is the most common, severe, and widely studied type of dystrophy in humans [167]. In patients with DMD, ATP and total adenosine are severely reduced in muscles (±50%). This decrease could be due to mitochondrial dysfunctions and an increased degradation of adenosine that is secreted by urine [168]. Treatments were carried out to prevent the loss of adenosine levels by adenylosuccinic acid (which increases cellular adenosine levels) or by inhibiting purine breakdown with allopurinol, leading to improved muscle strength and reduced lipid deposition [169].

## 7. Conclusions

In conclusion, the purinergic system is largely involved in the control of sarcopenia and muscle homeostasis. Adenosine A2A–A2B receptors play a fundamental role in muscle maintenance. Therapeutically, the activation of these receptors could prevent myogenic alterations and muscle loss in sarcopenia associated with aging and other pathologies. Nevertheless, more research is necessary to find new therapeutic strategies for secondary sarcopenia, including rheumatoid cachexia and other disorders.

## 8. Patents

A.M., M.M.-B., M.F., R.L., and G.H.-B. have filed a patent on the use of dipyridamole as a novel therapy for muscular myogenesis disorders and inflammatory arthritis.

## Data Availability

Not applicable.
